# Supplementary material for: A structural polymer for highly efficient all-day passive radiative cooling
Source: Nat Commun. 2021 Jan 14;12:365. doi: 10.1038/s41467-020-20646-7 (PMC7809060; doi:10.1038/s41467-020-20646-7)
Supplement: Supplementary file 1 — Supplementary Information [file 41467_2020_20646_MOESM1_ESM.pdf]

Supplementary Information

A Structural polymer for highly efficient all-day passive radiative cooling

Tong Wang, Yi Wu, Lan Shi, Xinhua Hu, Min Chen, Limin Wu*

*Corresponding author. Email: lmw@fudan.edu.cn (L. W.)

Supplementary note 1. Materials and Characterizations

PMMA (molecular weight, 150000), tetraethoxysilane (TEOS, ≥ 99%), ammonia solution ($NH_3 \cdot H_2O$, 28%), hydrofluoric acid (HF, 40%) and 1H,1H,2H,2H-perfluorooctyltrichlorosilane (PFOTS) were purchased from Aladdin Chemical Reagent Corp. Ethanol (EtOH, ≥ 99.7%) was provided by Shanghai Titan Scientific Co., Ltd. Acetone (≥ 99.5%) was obtained from Shanghai Dahe Chemical Co., Ltd. Polydimethylsiloxane (PDMS, SYLGARD 184) was received from Dow Corning Corp. The monodisperse SiO_2 microspheres (5 μm) were purchased from Zhiyi Microsphere Technology Co., Ltd. SiO_2 nanospheres (200 nm) were synthesized according to the sol-gel method and dispersed in acetone. All these materials were used as received without further purification. Deionized water was used throughout the experiment.

The morphologies of the monolayers SiO_2 templates and structural polymers were observed on a scanning electron microscope (Zeiss Gemini SEM 500) after gold coating, and elemental composition of PMMA/SiO_2 composite was examined by energy dispersive spectroscope (EDS) conducted on SEM. Thermogravimetric analysis (TGA, Q500, USA, TA Ltd) was performed to determine the residual amount of SiO_2 in the $PMMA_{HPA}$. Fourier transform infrared spectroscopy (FTIR, Nicolet 6700, Thermo Fisher Scientific, USA) was carried out to reveal the strong infrared absorbance between 770 and 1250 cm^{-1} (8-13 μm). The size distribution of pores in $PMMA_{HPA}$ was statistically identified by Nano Measurer. The photographs of the pristine PMMA, PMMA/SiO_2 composite and $PMMA_{HPA}$ films were taken with a digital camera (X-T20, Fujifilm, Japan). The porosity of $PMMA_{HPA}$ can be calculated using a density-based porosity measurement based on the following equations:

$$p_{PMMA_{HPA}} = \frac{v_1 - v_0}{v_1} \times 100\% \tag{1}$$

$$V = \frac{\mathrm{m} - m^*}{\rho_l} \tag{2}$$

Where p is the porosity of the $PMMA_{HPA}$ film, V_1 is the volume of the porous film and V_0 is the volume of the solid film. Here the volume (V) was measured by a densimeter (ethanol was used as the liquid medium). m is the mass of the film, m^* is the mass of the film immersed in the liquid and ρ_l is the density of the liquid medium. The porosity data were obtained by three independent measurements. The thickness at five positions for each sample was measured *via* a digital display micrometer (Mintel, Germany) with a precision of ± 1 μm. It is noteworthy that all the effective thicknesses in this article are 40% of the measured thicknesses due to the ~ 60% porosity of the $PMMA_{HPA}$ film. The water contact angles (WCAs) of the PMMA, $PMMA_{MPA}$, $PMMA_{NP}$ and $PMMA_{HPA}$ films were determined with an OCA15 contact angle analyzer (Dataphysics, Germany) to investigate the wetting property of the polymer surfaces. The average value of more than five parallel measurements on different sites was reported and the error bars represent the standard deviation. The durability of our $PMMA_{HPA}$ films under long-term UV radiation was carried out in a QUV accelerated weathering tester (QUV/se, Q-Panel Co., Ltd, USA) equipped with UVA-340 lamps at a wavelength of 310 nm. The samples were exposed to UV-irradiation (intensity of 0.71 W/m^2) at 60 °C for 4 h in dry conditions, followed by condensation at 50 °C for 4 h with UV lamps off.

Supplementary Note 2. Finite-Difference-Time-Domain Simulations

To investigate the optical mechanisms behind the structural PMMA films, Finite-Difference Time-Domain (FDTD) simulations were carried out using FDTD Solutions software (V8.19.1584, Lumerical Co. Ltd). Due to computational constraints of our computer, we used two dimensional (2D) models for numerical calculations. Plane wave was used as the light source and the injection light wavelength was from 0.3 to 25 μm. Periodic boundary was used along the x-axis and the perfect match layer (PML) boundary was used along the y-axis. Mesh size was set as 50 nm for thick PMMA and 10 nm for thin PMMA during FDTD simulations. Frequency-domain field monitor was used to monitor the reflectance and transmittance. Considering the non-polarized incident light in experiments, the reflectance results were calculated by averaging the results for transverse electric (TE) and transverse magnetic (TM) polarizations. In the LWIR (8-13 μm) region, we simulated the average emissivity at different incidence angles.

To investigate the influence of nanopore and micropore sizes on the reflectance performance, we established a random nanopores model with different sizes in the range of 50 to 500 nm and a periodic micropores model with different sizes in the range of 1 to 10 μm, respectively. We stipulated a determined thickness value (effectively ~ 20 μm) and an identical pore distribution density for each model. We also examined the effect of porosity (~ 5% to ~ 80%) on the reflectance performance and keep other parameters the same. When investigating the influence of the hierarchical porous structure on the optical properties, we designed four types of PMMA models and keep their effective thicknesses consistent. However, owing to the computational limits and overlong computing time, we simplified the structure models and reduced the effective thickness of four types of PMMA films to ~ 80 μm (~ 160 μm effective thickness was used in

experiments in Fig. 4). For example, it took us about four hours to compute one 2D-$PMMA_{HPA}$ model with effective thickness of ~ 80 μm.

Supplementary Note 3. Figures

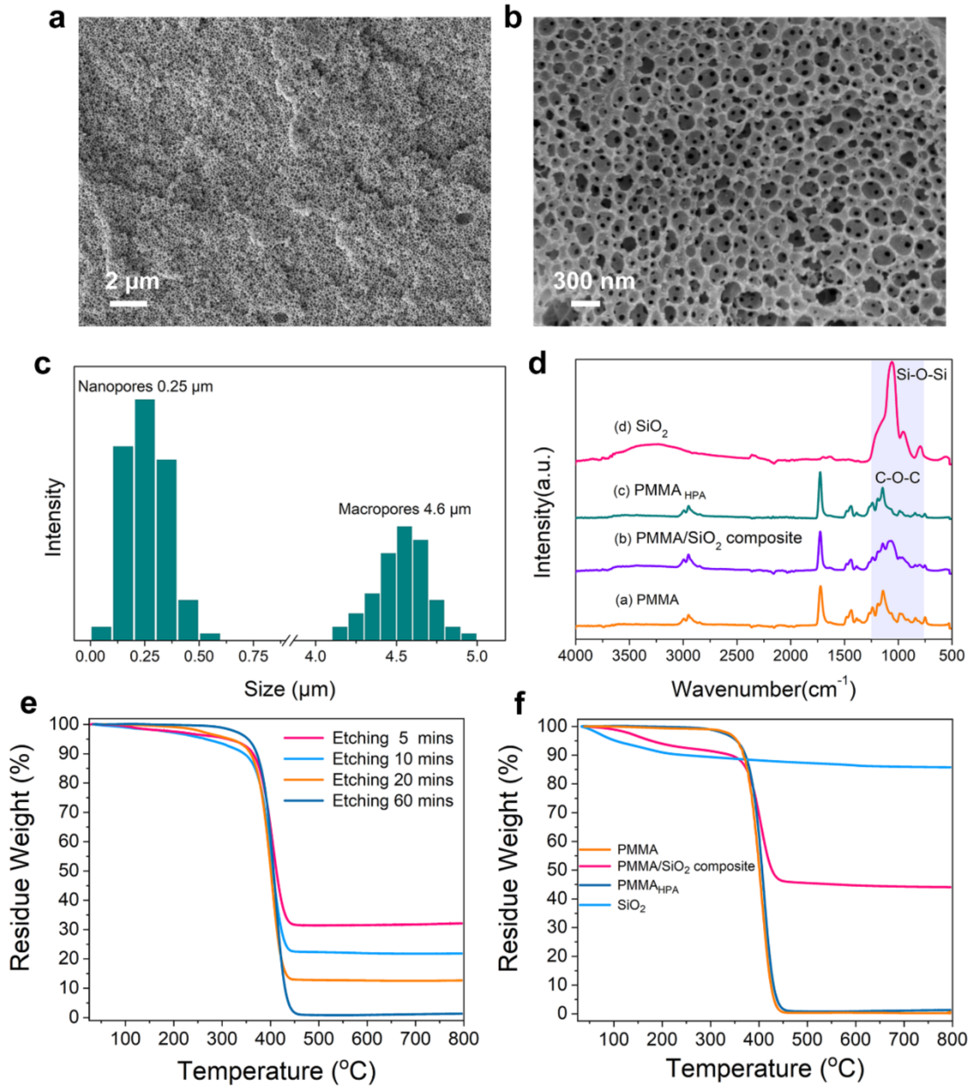

Supplementary Figure 1. Characterization of the $PMMA_{HPA}$ films. a, b, SEM micrographs of the cross-section of $PMMA_{HPA}$. **c,** Size distributions of nanopores and micropores in $PMMA_{HPA}$. **d,** FT-IR spectra of pristine PMMA, PMMA/SiO_2 composite, $PMMA_{HPA}$ and SiO_2 nanoparticles. The strong emission from 8 to 13 μm is mainly contributed by the complex infrared emission of C-O-C and Si-O-Si stretching vibrations between 770 and 1250 cm^{-1}. **e,** TGA analysis of the $PMMA_{HPA}$ film in different etching time. **f,** TGA analysis of pristine PMMA, PMMA/SiO_2 composite, $PMMA_{HPA}$ and SiO_2 nanoparticles.

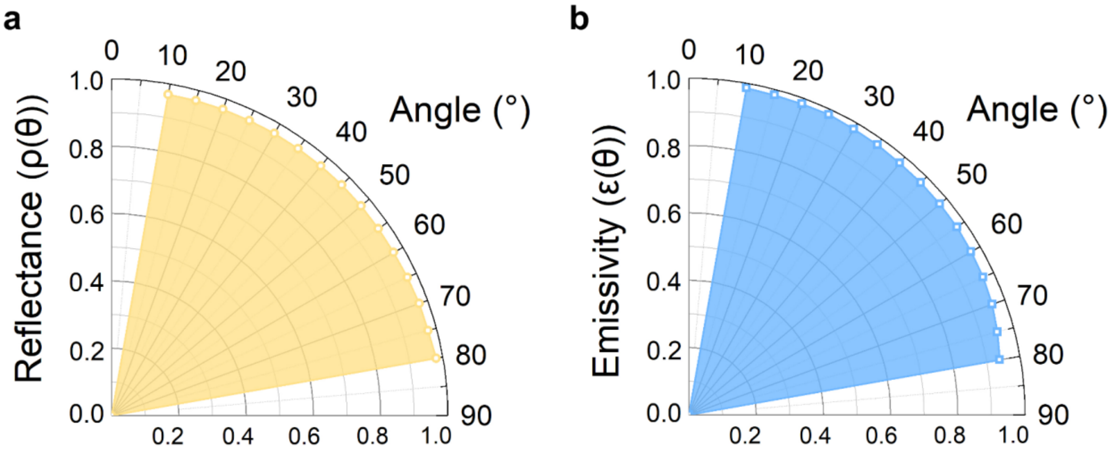

Supplementary Figure 2. $\overline{\rho}_{0.4-1.1\,\mu m}$ and $\overline{\varepsilon}_{LWIR}$ at different incidence angles. a, Measured average reflectance of the $PMMA_{HPA}$ film in the wavelength range of 0.4-1.1 μm at different incidence angles. **b,** Simulated average emissivity of the $PMMA_{HPA}$ film in the wavelength range of 8-13 μm at different incidence angles.

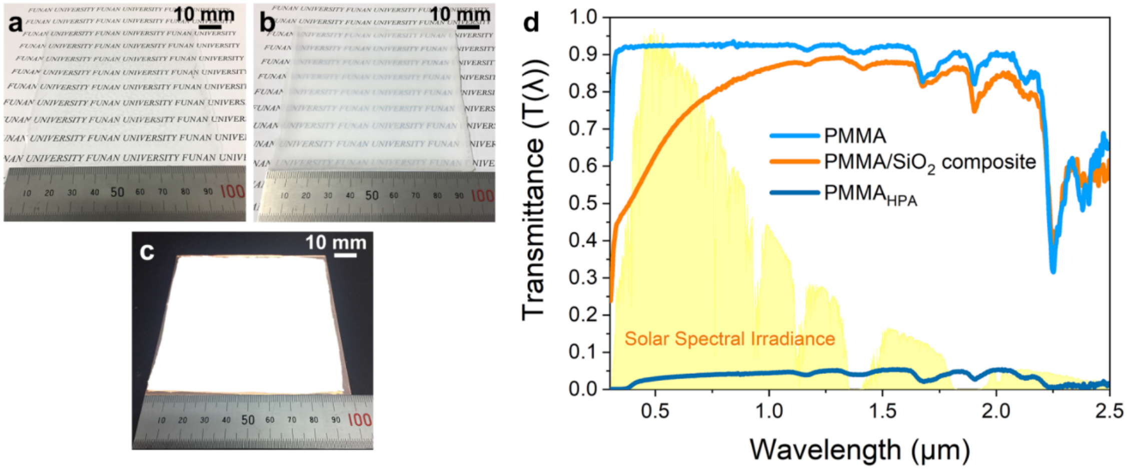

Supplementary Figure 3. Transmittance changes during the $PMMA_{HPA}$ preparation. a, Photo of pristine PMMA film. **b,** Photo of PMMA/SiO_2 composite film. **c,** Photo of $PMMA_{HPA}$ film. **d,** Spectral transmittance of the corresponding films in the range of solar spectrum from 0.3 to 2.5 μm.

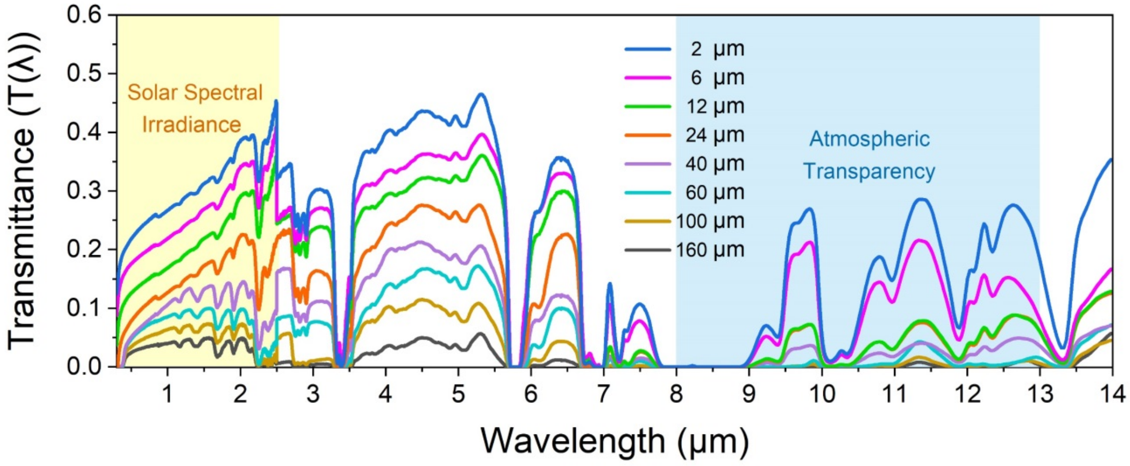

Supplementary Figure 4. Variation in the spectral transmittance of $PMMA_{HPA}$ films with effective thickness. As expected, transmittance drops with increasing thickness for the solar wavelengths.

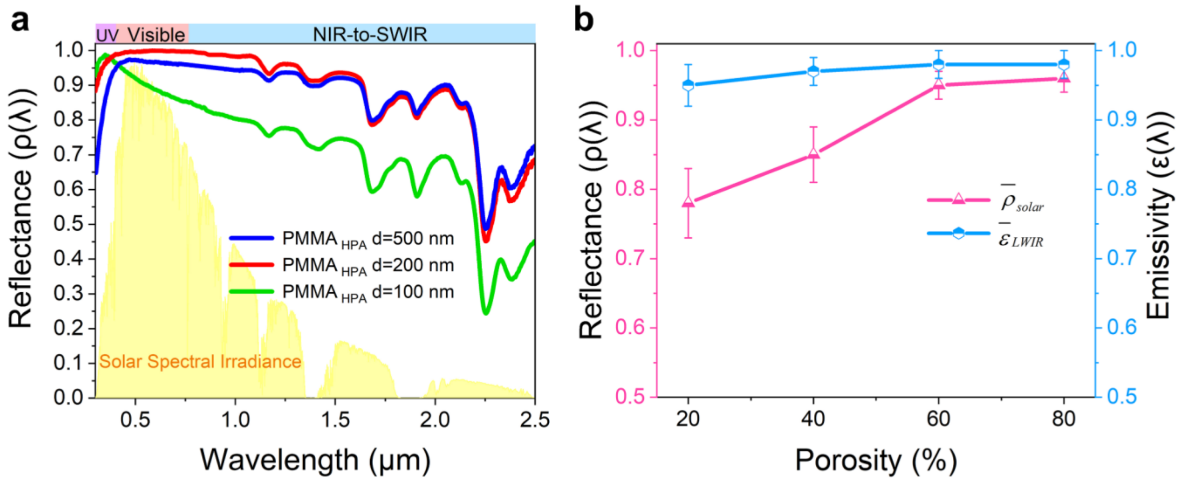

Supplementary Figure 5. Influence of nanopore sizes and porosity on the optical performance. a, Reflectance spectra of the $PMMA_{HPA}$ films with different nanopore sizes. SiO_2 nanospheres with diameter of ~ 100, 200 and 500 nm were synthesized by a sol-gel method and used as the nanopore templates. **b,** Variation in $\overline{\rho}_{solar}$ and $\overline{\varepsilon}_{LWIR}$ of the $PMMA_{HPA}$ films with porosity. The solar reflectance of $PMMA_{HPA}$ film rises with the increase of porosity while its thermal emittance does not change significantly. Both $\overline{\rho}_{solar}$ and $\overline{\varepsilon}_{LWIR}$ reach high values in ~ 60% porosity. The error bars represent the standard deviation.

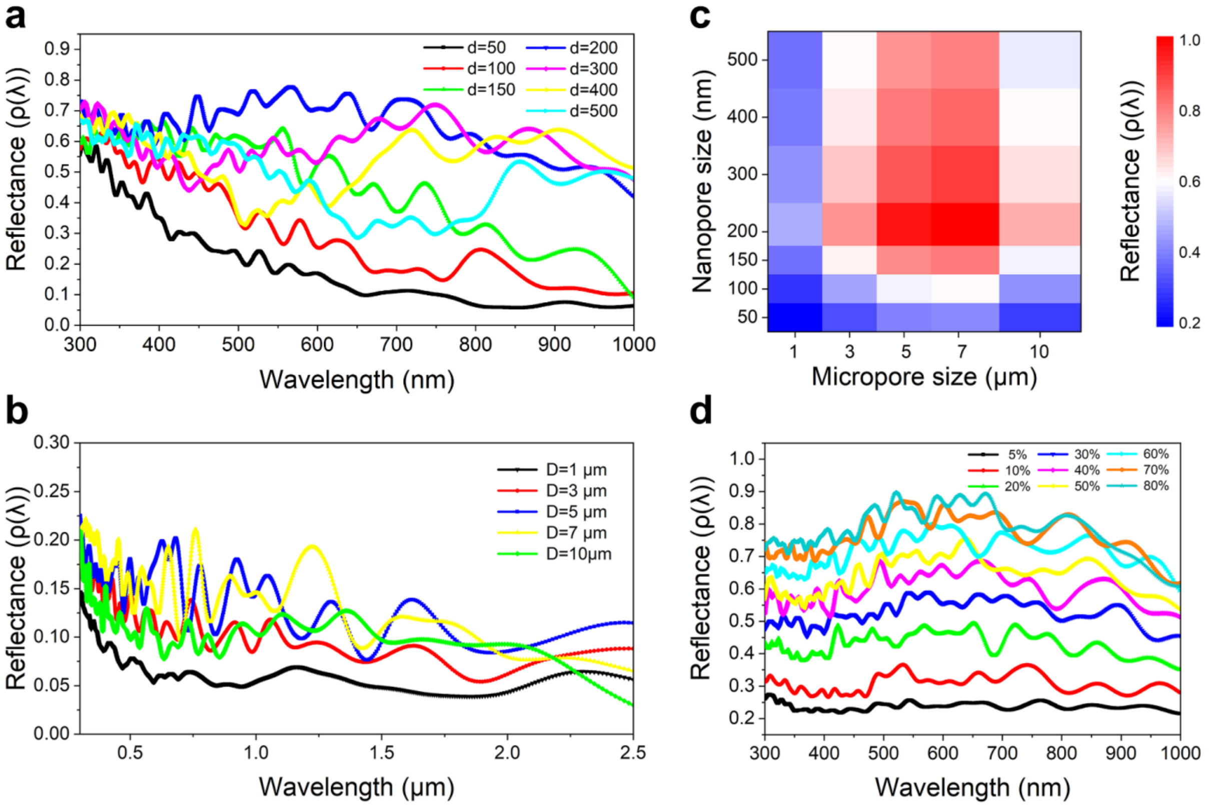

Supplementary Figure 6. Parameter optimization of pore sizes and porosity based on the FDTD simulations. a, Simulated reflectance of $PMMA_{NP}$ films with different nanopore sizes. **b,** Simulated reflectance of $PMMA_{MPA}$ films with different monolayer micropore sizes. **c,** Normalized average reflectance as a function of varied nanopore and micropore sizes. **d,** Simulated reflectance of PMMA films with different porosities ranging from 5% to 80%.

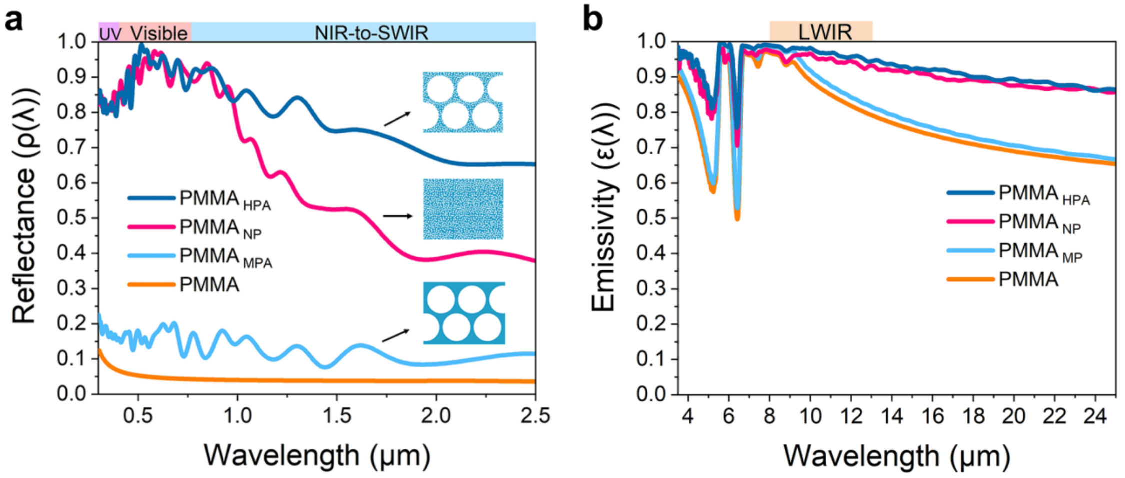

Supplementary Figure 7. Simulated reflectance and emissivity spectra of four types of PMMA films. a, Simulated reflectance spectra across the solar wavelengths (inset, the surface view of each model used in the simulations). **b,** Simulated infrared emissivity spectra. We kept the effective thickness of each model consistent (effectively ~ 80 μm).

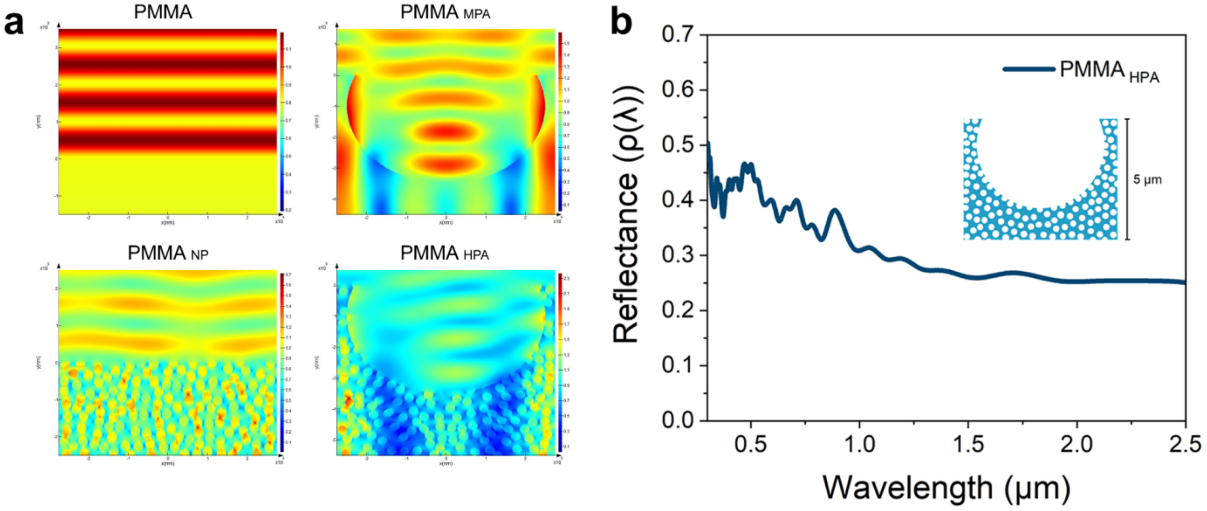

Supplementary Figure 8. FDTD simulations of the PMMA films with thickness of ~ 5 μm. a, Electric field distributions of four types of PMMA films at wavelength of ~ 2 μm. **b,** Simulated reflectance spectra across the solar wavelengths of the $PMMA_{HPA}$ film with thickness of ~ 5 μm (inset, the cross-section view of $PMMA_{HPA}$ model).

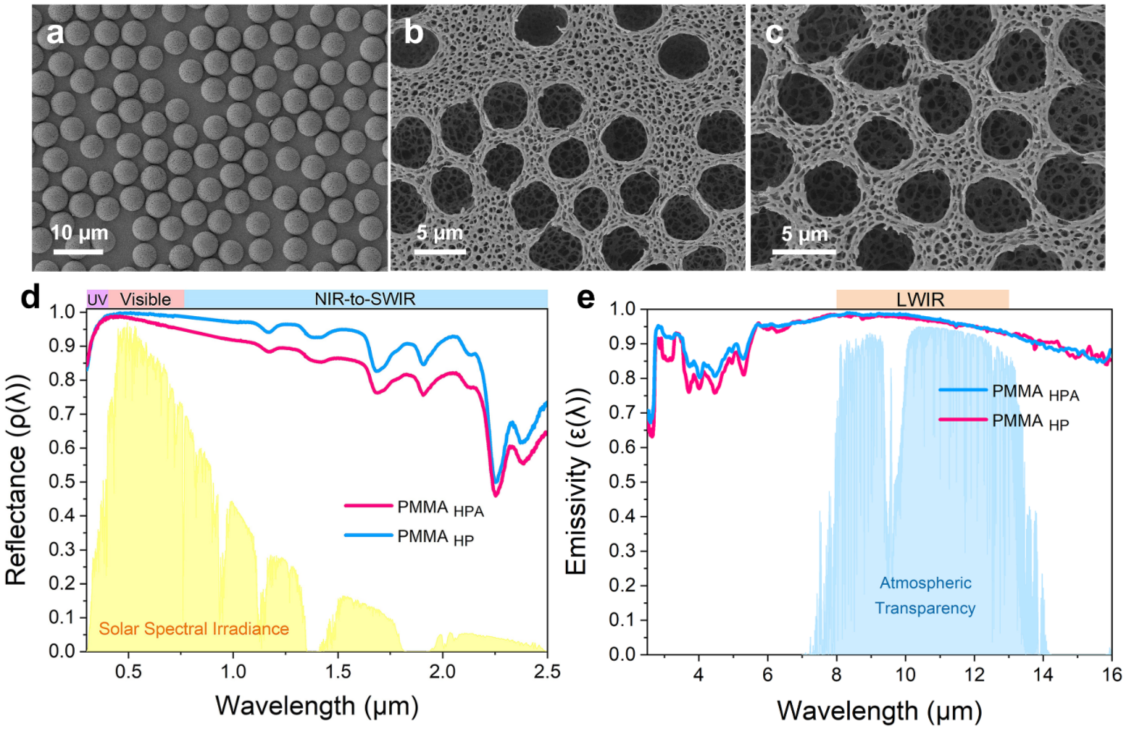

Supplementary Figure 9. SEM micrographs and optical properties of $PMMA_{HPA}$ and $PMMA_{HP}$ films. a, SEM micrograph of random loose-packed monolayer SiO_2 templates. **b, c,** SEM micrographs of $PMMA_{HP}$ film fabricated using (**a**) as templates, showing loose random micropores made of randomized nanopores. **d,** Reflectance spectra across the solar wavelengths of $PMMA_{HPA}$ and $PMMA_{HP}$ films. **e,** Infrared emissivity spectra of $PMMA_{HPA}$ and $PMMA_{HP}$ films.

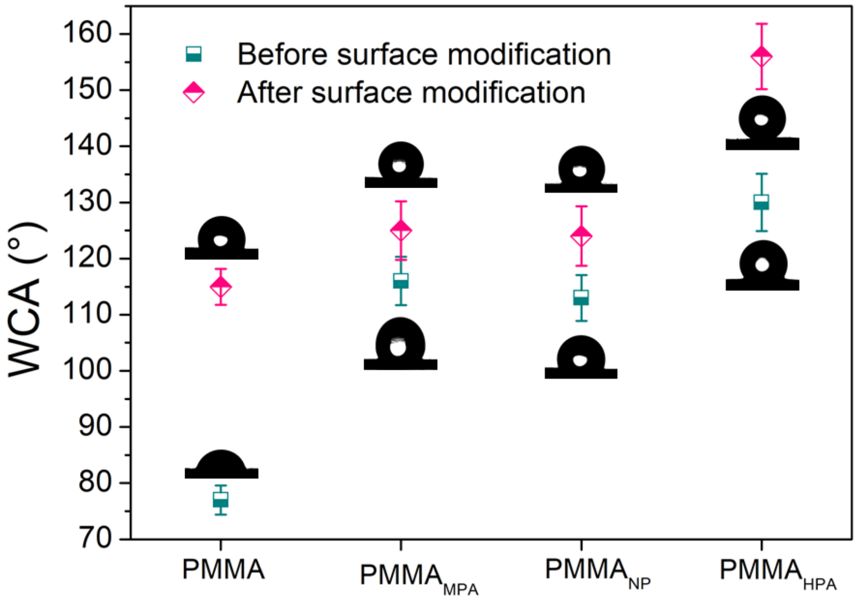

Supplementary Figure 10. WCA variations of PMMA, PMMA$_{MPA}$, PMMA$_{NP}$, PMMA$_{HPA}$ films after surface modification. After modification by fluorosilane treatment, the WCAs of the four types of PMMA films were increased, but only the PMMA$_{HPA}$ film reached superhydrophobic state, with a water contact angle of ~156°. The error bars represent the standard deviation.

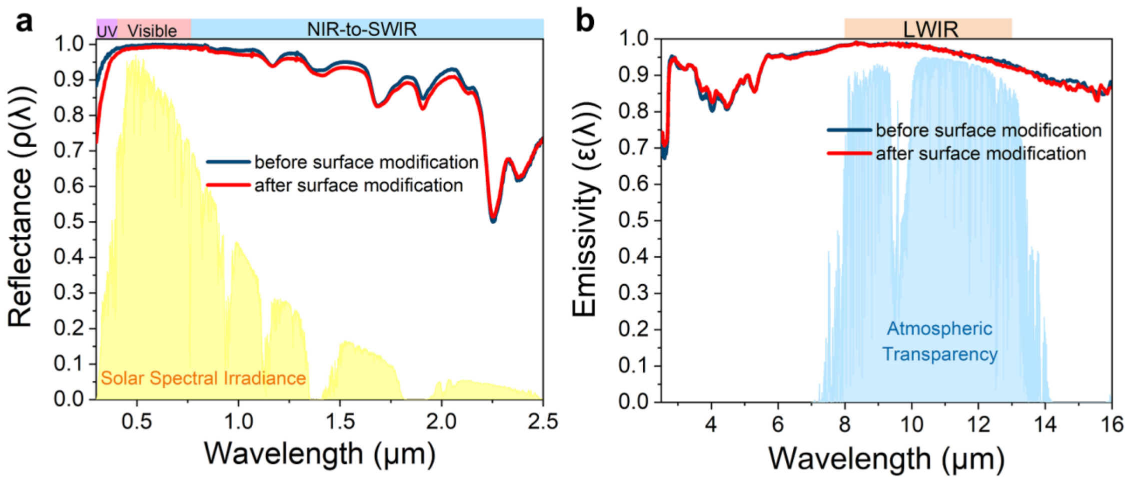

Supplementary Figure 11. Spectral reflectance and emissivity of the $PMMA_{HPA}$ film before and after the surface modification by fluorosilane.

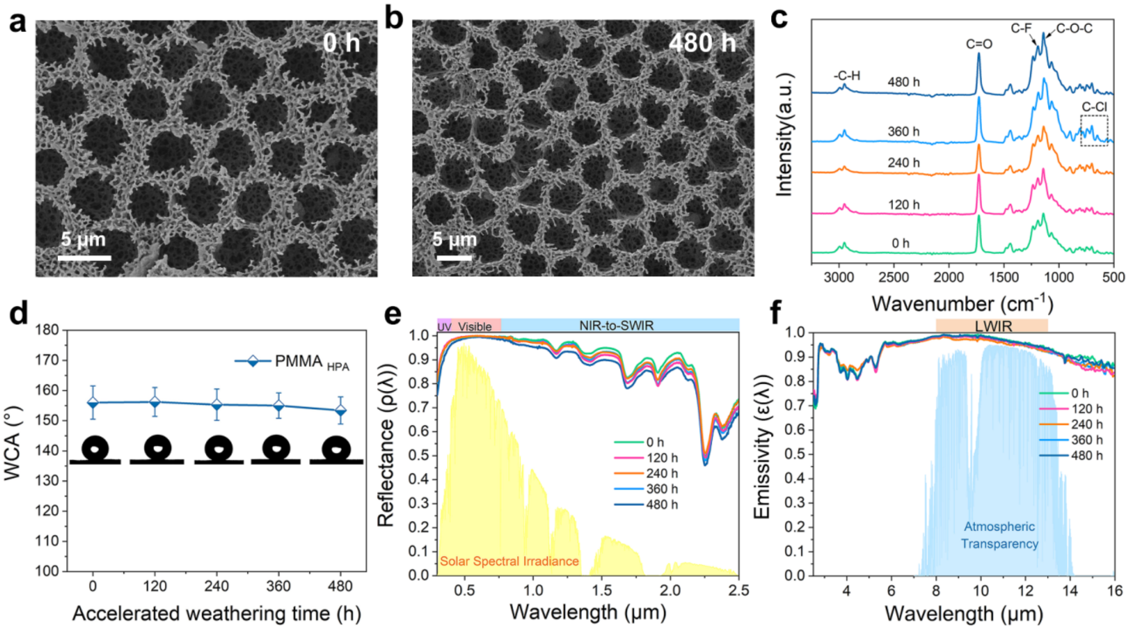

Supplementary Figure 12. Characterization of the surface-modified PMMA$_{HPA}$ films before and after UV accelerated weathering treatment. a, b, SEM micrographs of PMMA$_{HPA}$ films before and after accelerated weathering treatment for 480 h. **c, d,** FT-IR spectra and WCA variations of the PMMA$_{HPA}$ films with the accelerated weathering time. **e, f,** Reflectance and emissivity spectra of the PMMA$_{HPA}$ films with the accelerated weathering time.

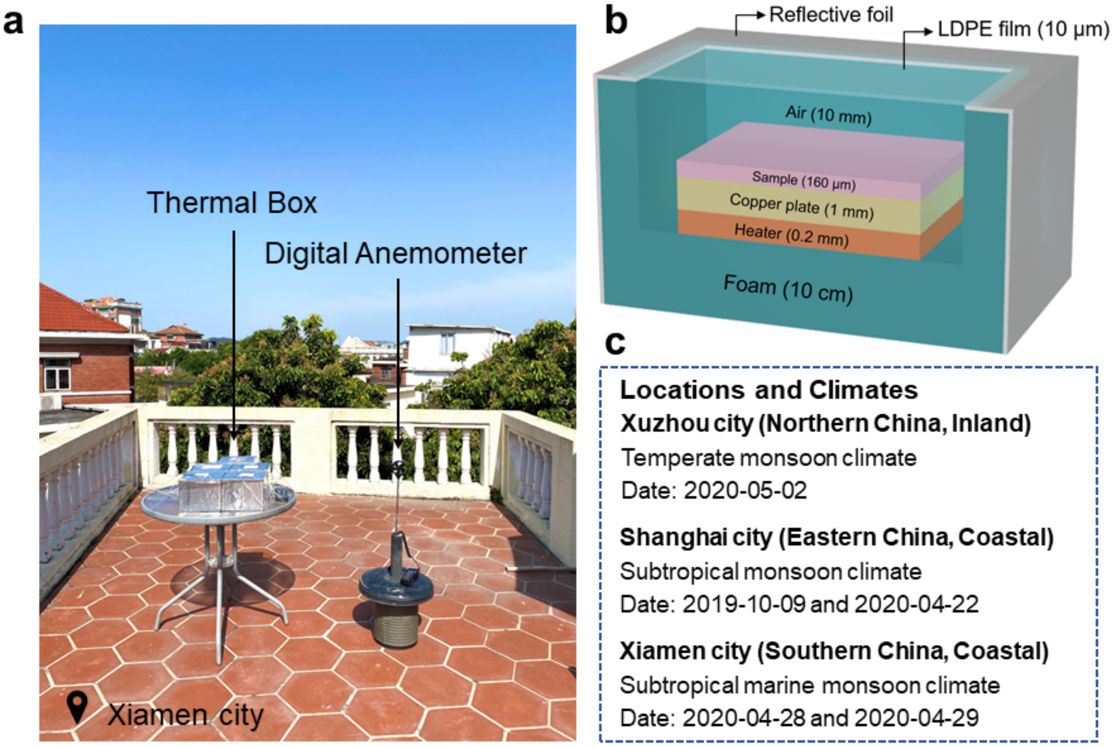

Supplementary Figure 13. Passive daytime radiative cooling performance measurements under clear skies in different locations and climates. a, Photo of the experimental apparatus on the rooftop in Xiamen city, China, on April 28, 2020. **b,** Cut-out schematic of the thermal box apparatus with a feedback-controlled heater. The heater kept the same $PMMA_{HPA}$ surface temperature as that of the ambient environment, minimizing convective and conductive heat losses. The heater and copper plate were removed to demonstrate the subambient cooling performance. **c,** Locations and climates information of the three cities, showing three types of climates. The local temperatures of the above cities and various dates can be seen from meteorological station: https://www.wunderground.com/history/daily/cn/xuzhou/ZSXZ/date/2020-5-2, https://www.wunderground.com/history/daily/cn/shanghai/ZSSS/date/2019-10-9, https://www.wunderground.com/history/daily/cn/shanghai/ZSSS/date/2020-4-22, https://www.wunderground.com/history/daily/cn/xiamen/ZSAM/date/2020-4-28, https://www.wunderground.com/history/daily/cn/xiamen/ZSAM/date/2020-4-29.

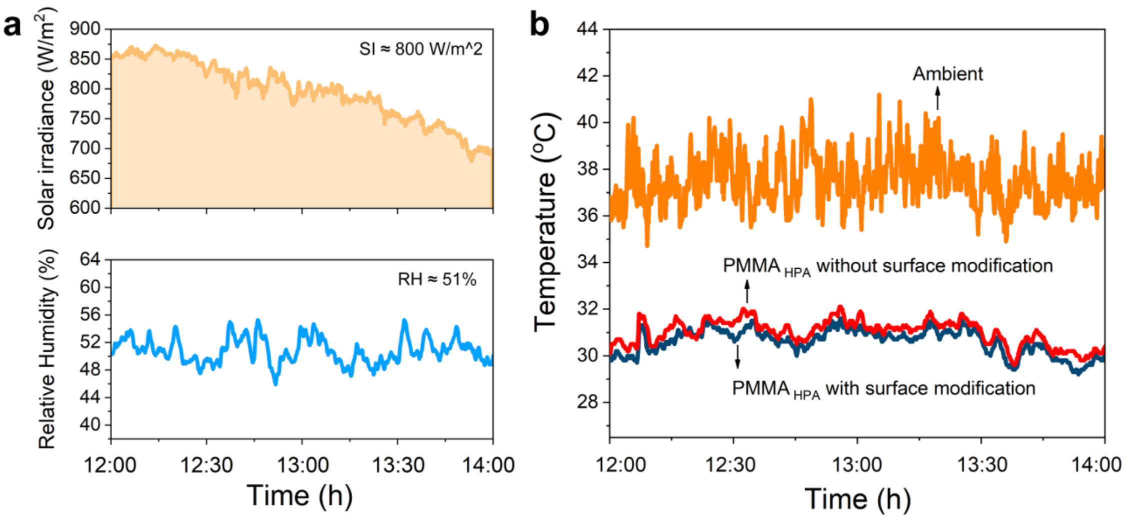

Supplementary Figure 14. Effect of the surface modification on the daytime radiative cooling performance of the PMMA$_{HPA}$ film. a, Solar irradiance and relative humidity on August 19, 2020, in Shanghai city. **b,** Temperature tracking of the air and PMMA$_{HPA}$ films with and without surface modification.

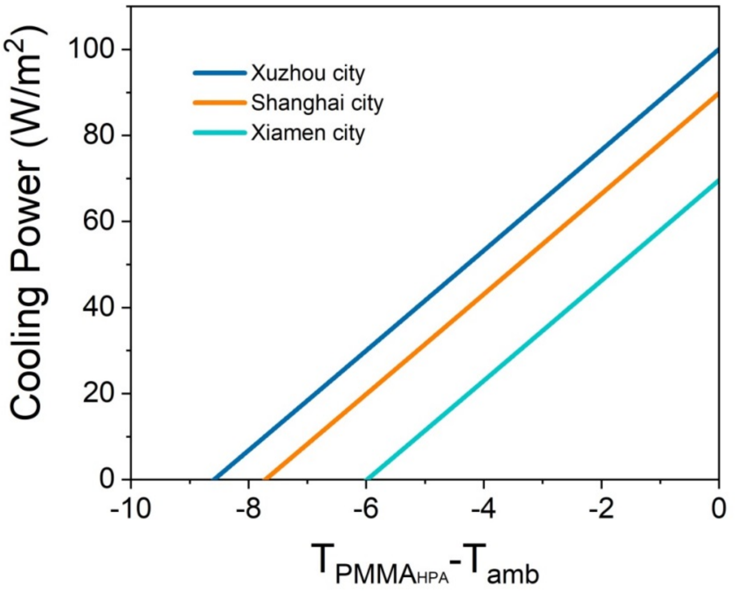

Supplementary Figure 15. Calculated net cooling power during daytime in different locations. The nonradiative heat coefficient h_c = 3 W/m^2/K was used in the calculations.

Supplementary Tables.

Table S1 | Results of the exposure tests of $PMMA_{HPA}$ films in Shanghai city.

Days	Average temperature (°C)	Average relative humidity (%)	Average solar intensity (W/m^2)	Solar reflectance	Thermal emittance	WCA (°)
05-14 August, 2020	31.2	75.2	830	0.95	0.98	156
15 -24 August, 2020	32.8	74.8	805	0.95	0.98	155
25 August-03 September, 2020	29.6	76.3	790	0.94	0.98	155
04-13 September, 2020	27.1	78.5	755	0.94	0.98	154

Average solar intensity during midday in sunny day excluding occasional rainy day.

Table S2 | Comparison of PDRC performances of the radiative coolers.

**Materials**	**Structure**	**Solar reflectance/ thermal emittance**	**Solar intensity (W/m^2)**	**Relative humidity (%)**	**Temperature drop (°C)**	**Cooling power (W/m^2)**	**Year**
**This work**	**micropore array/random nanopores**	**0.95/0.98**	**860**<br>**930**	**38**<br>**64**	**8.9**<br>**5.5**	**85**	**2020**
PDMS/Al_2O_3 (*1*)	photonic structure	0.95/0.96	862	22.7	5.1	90.8	2020
PVDF/TEOS /SiO_2 (*2*)	photonic structure	0.97/0.96	< 1000	-	< 6	61	2019
Wood (*3*)	mechanical pressed and porous structure	0.96/-	< 750	*	~ 4	16	2019
PDMS/metal (*4*)	-	-/-	< 853.5	35	~ 11	120	2019
P(VdF-HFP) (*5*)	hierarchical pores	0.96/0.97	890	dry	~ 6	96	2018
TPX/SiO_2/Ag (*6*)	hybrid metamaterial	-/0.93	900	*	-	93	2017
SiO_2/HfO_2/Ag (*7*)	photonic structure	0.97/-	850	*	4.9	40.1	2014

* no related test was shown in the papers.

- means no data were shown in the papers.

Supplementary Reference

1. Zhang, H. et al. Biologically inspired flexible photonic films for efficient passive radiative cooling. *P. Natl. Acad. Sci. USA.* **117**:202001802 (2020).
2. Wang, X. et al. Scalable flexible hybrid membranes with photonic structures for daytime radiative cooling. *Adv. Funct. Mater.* **30**, 1907562 (2019).
3. Li, T. et al. A radiative cooling structural material. *Science* **364**, 760-763 (2019).
4. Zhou, L. et al. A polydimethylsiloxane-coated metal structure for all-day radiative cooling. *Nat. Sustain.* **2**, 718-724 (2019).
5. Mandal, J. et al. Hierarchically porous polymer coatings for highly efficient passive daytime radiative cooling. *Science* **362**, 315-319 (2018).
6. Zhai, Y. et al. Scalable-manufactured randomized glass-polymer hybrid metamaterial for daytime radiative cooling. *Science* **355**, 1062-1066 (2017).
7. Raman, A. P., Anoma, M. A., Zhu, L., Rephaeli, E. & Fan, S. Passive radiative cooling below ambient air temperature under direct sunlight. *Nature* **515**, 540-544 (2014).
